# Enjoyment for High-Intensity Interval Exercise Increases during the First Six Weeks of Training: Implications for Promoting Exercise Adherence in Sedentary Adults

**DOI:** 10.1371/journal.pone.0168534

**Published:** 2016-12-14

**Authors:** Jennifer J. Heisz, Mary Grace M. Tejada, Emily M. Paolucci, Cameron Muir

**Affiliations:** 1 Department of Kinesiology, McMaster University, Hamilton, Ontario, Canada; 2 Department of Psychology, Centre for Neuroscience, Brock University, St. Catherines, Ontario, Canada; University of Alabama at Birmingham, UNITED STATES

## Abstract

This is the first study to show that enjoyment for high-intensity interval exercise increases with chronic training. Prior acute studies typically report high-intensity interval training (HIT) as being more enjoyable than moderate continuous training (MCT) unless the high-intensity intervals are too strenuous or difficult to complete. It follows that exercise competency may be a critical factor contributing to the enjoyment of HIT, and therefore building competency through chronic training may be one way to increase its enjoyment. To test this, we randomly assigned sedentary young adults to six weeks of HIT or MCT, and tracked changes in their enjoyment for the exercise. Enjoyment for HIT increased with training whereas enjoyment for MCT remained constant and lower. Changes in exercise enjoyment were predicted by increases in workload, suggesting that strength adaptions may be important for promoting exercise enjoyment. The results point to HIT as a promising protocol for promoting exercise enjoyment and adherence in sedentary young adults.

## Introduction

The physical benefits of exercise are widely known, yet half of the adult population are not sufficiently active for good health [1]. For sedentary individuals, a key barrier to starting an exercise program is the preconceived notion that exercising is not enjoyable [2] and failing to find enjoyment from exercise can make it more difficult to adhere to an exercise program over time [3]. Accumulating evidence suggests that an *acute* bout of high-intensity interval exercise may be more enjoyable than an acute bout of traditional forms of continuous exercise [4–6]. However, it is unclear how the enjoyment of high-intensity interval exercise changes over time with *chronic* training. The present study examined the changes in enjoyment of high-intensity interval training (HIT) versus moderate continuous training (MCT) over a six-week period in sedentary younger adults.

HIT consists of short high-intensity intervals interspersed with lower-intensity recovery intervals. Compared to MCT, HIT induces similar-to-greater improvements in fitness and cardiovascular function but in a shorter amount of time [7]. An acute bout of HIT can also be more enjoyable than an acute bout of moderate continuous exercise [4, 5]. Importantly, sedentary young adults report greater enjoyment from a single bout of HIT and endorsed it as an exercise regime they would chose to continue on their own [5].

That said, there are a multitude of HIT protocols and not all of them elicit the same level of enjoyment. Protocols with 120s high-intensity intervals are rated as being less enjoyable than protocols with 30s or 60s high-intensity intervals [8]. Furthermore, one study found that an acute bout of HIT was equally enjoyable to an acute bout of moderate continuous exercise but induced higher levels of negative feelings and fatigue immediately following, and only half of the participants were able to successfully complete the high-intensity bout [9]. One aspect reducing enjoyment of these more strenuous protocols may be related to the individual’s ability, or competency, to successfully complete the exercise. It follows that building competency at HIT through training may increase enjoyment for the exercise over time. Indeed, positive feedback from successfully completing a difficult task increases feelings of competency [10] and enjoyment [11]. Alternatively, the accumulated fatigue or physical stress from chronically performing a strenuous exercise may actually increase negative feelings and reduce enjoyment for the exercise over time.

The present study is the first to examine changes in the enjoyment of HIT versus MCT over the first six-week of an exercise program in sedentary young adults. Based on previous literature examining acute bouts, we expected initial ratings of HIT to be at least as enjoyable as MCT [4, 5]; however, it was unclear how enjoyment for HIT would change during the following weeks of exercise adoption when both the behavioral and physiological changes are most abrupt [12]. As an exploratory analysis, we assessed whether training-related changes in exercise enjoyment would be predicted by changes in exercise competency, as indexed by workload and aerobic fitness.

## Materials and Methods

### Participants

Forty healthy young adults were recruited from McMaster University. Hamilton Integrated Research Ethics Board Approved this study. Project Number: 0147. All participants provided written informed consent and were compensated for the time in the study.

To ensure a similar number of males and females in each group, participants were stratified by sex and then randomly assigned to one of two groups: a moderate intensity continuous training (MCT) group or a high-intensity interval training (HIT) group; two females from the HIT group did not complete the training (one due to a scheduling conflict and the other due to a noxious response during the first exercise session) and thus, their data was excluded from the analyses. Two additional participants from the HIT group (one female and one male) were excluded from all analyses because of missing data on the key measure of exercise enjoyment. Table 1 presents the demographic information for the participants included in the analyses.

**Table 1:** Participant demographics and mean activity levels three-months prior to the start of training.

	HIT	MCT
N	17	19
Sex	12 females; 5 males	13 females; 6 males
Age	21.4 (2.9)	20.4 (1.3)
**Prior aerobic exercises**		
Frequency (hours/week)	0.8 (1.0)	0.6 (0.7)
Intensity (max 7)	0.7 (1.1)	0.6 (1.0)
**Prior resistance training**		
Frequency (hours/week)	0.2 (0.4)	0.1 (0.2)
Intensity (max 7)	0.6 (1.1)	0.3 (0.8)

Standard deviations in parenthesis

All participants were students who met the inclusion criteria of engaging in no more than one hour of vigorous physical activity per week. To determine this, participants reported their average weekly engagement in aerobic and resistance training exercises for the three-month prior to the start of the experiment by answering questions about frequency (hours/week) and intensity (0–7, where 7 was the maximum intensity and participants who did not engage in the exercise reported 0). Three-month prior to the start of the experiment, both HIT and MCT groups engaged in similarly low levels of aerobic and resistance training exercises and at low intensities. Independent samples *t* tests confirmed no group differences in baseline physical activity levels (all *p* > .05; Table 1).

### Procedure

#### Pre-testing

Participants were trained in one of two training periods: the first took place from October to December and the second took place from February to April. To control for potential seasonal effects, participants trained during each time period were assigned to a group following the randomization scheme described above. Prior to training, all participants completed a maximal aerobic fitness test on a cycle ergometer (Lode, Groningen, Netherlands) using a metabolic cart to determine VO_2_ peak (Medisoft Ergocard). VO_2_ peak, peak heart rate (HR) and peak power output (PPO) from this test were used to assess baseline fitness. Peak HR and PPO were used to create the exercise prescription for each participant. Body mass and height were measured to assess body mass index (BMI).

Resting-state/morning cortisol was also assessed. 12h-fasted saliva samples were collected in the morning using Salivettes®. Saliva samples were stored at -20°C until analysis. Saliva was centrifuged at 3000xg for 15 minutes and only the supernatant was assayed. All enzyme immunoassays were carried out on NUNC Maxisorb plates. Cortisol antibodies (R4866) and corresponding horseradish peroxidase conjugate were obtained from C. Munro of the Clinical Endocrinology Laboratory, University of California, Davis. Steroid standards were obtained from Steraloids, Inc. Newport, Rhode Island. Plates were first coated with 50μl of antibody stock diluted at 1:8500 in a coating buffer (50 mmol/L bicarbonate buffer pH 9.6). Plates were sealed and stored for 12–14 h at 4°C. 50μl wash solution (0.15 mol/L NaCl solution containing 0.5 ml of Tween 20/L) was added to each well to rinse away any unbound antibody, then 50μl phosphate buffer per well was added. The plates were incubated at room temperature for 2 hours before adding standards, samples, or controls. For each hormone, two quality control salivary samples at 30% and 70% binding (the low and high ends of the sensitive range of the standard curve) were prepared. 50μl cortisol horseradish peroxidase conjugate was added to each well, with 50μl of standard, sample, or control. After plate loading, plates remained incubated for 1 hour. Next, the plates were washed with 50μl wash solution and 100μl of a substrate solution of citrate buffer, H2O2 and 2,2’-azino-bis [3-ethylbenzthiazoline-6-sulfonic acid] was added to each well and the plates were covered and incubated while shaking at room temperature for 30–60 minutes. The plates were then read with a single filter at 405nm on the microplate reader (Titertek multiskan MCC/340). Blank absorbances were obtained, standard curves generated, a regression line was fit to the sensitive range of the standard curve (typically 40–60% binding) and samples were interpolated into the equation to get a value in pg per well. Each sample was assayed in duplicate and averages were used. Interplate variation (CV) was 6.45% while intraplate variation was 6.51%.

#### Exercise training

Supervised exercise training sessions were completed on a cycle ergometer (Lifecycle 95Ci) approximately three times per week (typically Monday, Wednesday and Friday) for six consecutive weeks. If a session was missed, it was made up that week or the following week. Participants performed each exercise session in a group, including a maximum of three participants per session. The composition of each session was based on the participant’s availability and so it was possible for participants from HIT and MCT groups to exercise at the same time.

The HIT protocol consisted of ten bouts of one-minute high-intensity interval to elicit ~90–95% peak HR followed by one-minute recovery interval at 30% PPO (min 50W) for a total of 20min. The MCT protocol consisted of a continuous bout of moderate intensity exercise to elicit ~70–75% peak HR for a duration of 27.5min to equate the energy expenditure with the HIT protocol. Both protocols included a 3min warm-up and 2min cool-down at 50W. Workload, HR, and Borg’s Rating of Perceived Exertion (RPE, scale 6 to 20) [13] were recorded throughout each exercise session, every minute for HIT and every other minute for MCT; analysis for HIT only consisted of the high-intensity intervals. Over the six weeks of training, workload was adjusted to elicit the target HR.

At the same time each week, immediately after Friday’s exercise session, participants completed the 18-item Physical Activity Enjoyment Scale (PACES) [14] in which they rated their enjoyment for the exercise that week on a seven-point scale. The maximum score is 126, with higher scores indicating greater enjoyment. The time and day that PACES was administered was kept constant to minimize the impact of extraneous factors on enjoyment ratings (e.g., a test the following day); however, this meant that participants who did not attend the Friday session did not complete PACES for that week.

#### Post-testing

After six weeks of training, all participants completed a second maximal aerobic test (identical to the pre-training test) to evaluate changes in VO2 peak, PPO and peak HR. Body mass was measured and BMI was recalculated. Resting-state cortisol was assessed as described above.

### Statistical Analysis

Data were initially screened for outliers, which were replaced with the next highest score in the data set that was not an outlier [15].

#### Manipulation checks

To confirm that the groups did not differ at baseline, one-way analysis of variance (ANOVA) were conducted on pre-training values for age, sex, VO_2_ peak, PPO, peak HR, body mass, BMI, and resting-state/morning cortisol. Welch test [15] was used to analyze body mass and BMI because these measures did not conform to the homogeneity of variance assumption. Resting-state/morning cortisol was not normally distributed; a natural logarithm transformation was done before analysis, and prior to the transformation the three missing values during the pre-test (2 from the HIT group and 1 from the MCT group) were replaced with the mean (8 ng/ml).

To verify that the exercise training induced aerobic fitness changes, we conducted two analyses. The first analysis examined the training data. Mean changes per week in workload, HR, and RPE during training were assessed using a mixed-model ANOVA with a within-subjects factor of week (1 to 6) and a between-subjects factor of group (HIT, MCT). The second analysis examined the changes in VO_2_ peak, PPO and peak HR from pre and post training tests using a mixed-model ANOVA with a within-subjects factor of test (pre, post) and a between-subjects factor of group (HIT, MCT). As a secondary analysis to provide information on other physiological adaptations that may have resulted from the exercise training, we also examined pre-to-post changes in body mass, BMI, and resting-state/morning cortisol.

#### Group differences in exercise enjoyment

Change in exercise enjoyment across the six weeks of training was assessed using a mixed model ANOVA with a within-subjects factor of week (1 to 6) and a between-subjects factor of group (HIT, MCT). Planned independent samples *t* tests were also used to examine whether HIT was more enjoyable than MCT at each week. Fourteen percent of the scores were missing at completely random (MCAR, *p* > 0.05). Missing scores for the first week were replaced with the group mean for that week [16] and missing scores for the remaining weeks were replaced with the last observation carried forward. Participants who completed more than two PACES were used in the analysis; as noted above, two participants (one male and one female) from the HIT group were excluded from all analyses because they did not meet this criterion.

To determine whether increases in exercise enjoyment were driven by exercise competency, we conducted a hierarchical linear regression analysis with a dependent variable of change in exercise enjoyment from weeks one to six. In step one of the model, age and sex were entered as independent variables to control for individual differences. Then, indices of exercise competence, including change in average training workload from weeks one to six as well as post-minus-pre changes in VO_2_ peak and PPO, were entered stepwise as independent variables.

## Results

### Manipulation checks

At baseline, groups did not differ with respect to age, sex, VO_2_ peak, PPO, peak HR, body mass, BMI, or resting-state cortisol (all *p* > .05; Table 2). During training, HIT elicited higher workload, HR, and RPE than MCT (all *p* < .001; Table 3). Workload increased linearly across the six weeks of training for both groups [main effect of week, *F*(5, 170) = 34.52, *p* < .001, *η*_*p*_^*2*^ = .50; linear contrast, *F*(1, 34) = 48.60, *p* < .001, *η*_*p*_^*2*^ = .59] but to a greater extent for HIT than MCT [group x week interaction, *F*(5, 170) = 5.40, *p* < .001, *η*_*p*_^*2*^ = .14; group differences in workload change, *t*(34) = 2.61, *p* < .05, *d* = .8]. There was also a main effect of week for HR [*F*(5, 170) = 4.33, *p* < .01, *η*_*p*_^*2*^ = .11] and a group x week interaction for RPE [*F*(5, 170) = 3.11, *p* < .05, *η*_*p*_^*2*^ = .08], however, this was driven by subtle variations from week to week that were within the desired target range. Pre-to-post training changes on the maximal aerobic test revealed increases in VO_2_ peak [main effect of pre/post: *F*(1, 34) = 17.93, *p* < .001, *η*_*p*_^*2*^ = .35] and PPO [main effect of pre/post: *F*(1, 34) = 57.69, *p* < .001, *η*_*p*_^*2*^ = .63] but no effect or interaction of group (Table 2). Body mass, BMI and resting-state/morning cortisol did not significantly differ between groups and were not affected by either training protocol (all *p* > .05).

**Table 2:** Means (standard errors) for pre and post-training assessments for the high-intensity interval training (HIT) and moderate continuous training (MCT) groups.

	HIT		MCT	
	Pre	Post	Pre	Post
VO_2_ peak (ml/kg/min)	31.8 (1.6)	35.8 (1.8)	30.2 (1.5)	33.1 (1.7)
PPO (W)	184 (11)	204 (12)	185 (10)	203 (11)
Peak HR (beats/min)	183 (3)	186 (2)	188 (2)	188 (2)
Body mass (kg)	58.0 (2.3)	58.6 (2.4)	64.9 (3.2)	64.7 (3.2)
BMI (kg/m^2^)	21.1 (0.5)	21.3 (0.5)	23.0 (1.0)	22.9 (1.0)
Cortisol (ng/ml)	8.6 (1.0)	7.5 (0.8)	8.3 (1.1)	6.5 (0.6)

Body mass index (BMI); Heart rate (HR); Peak power output (PPO); Watts (W)

**Table 3:** Means (standard errors) of training outcomes averaged across session and week for high-intensity interval training (HIT) and moderate continuous training (MCT).

	HIT						MCT					
	1	2	3	4	5	6	1	2	3	4	5	6
Workload	143 (8)	147 (8)	157 (9)	161 (10)	167 (10)	170 (11)	77 (7)	80 (8)	83 (9)	86 (10)	88 (10)	89 (10)
HR	175 (3)	174 (2)	174 (2)	175 (2)	172 (2)	176 (2)	146 (2)	142 (2)	139 (1)	140 (1)	140 (2)	142 (2)
RPE	15 (.3)	15 (.4)	15 (.4)	15 (.3)	15 (.3)	16 (.3)	11 (.3)	11 (.3)	11 (.4)	11 (.3)	11 (.3)	11(.3)

Heart rate (HR; beats/min); Rating of perceived exertion (RPE; scale 6–20); Workload in Watts

### Group differences in exercise enjoyment

**Fig 1:**
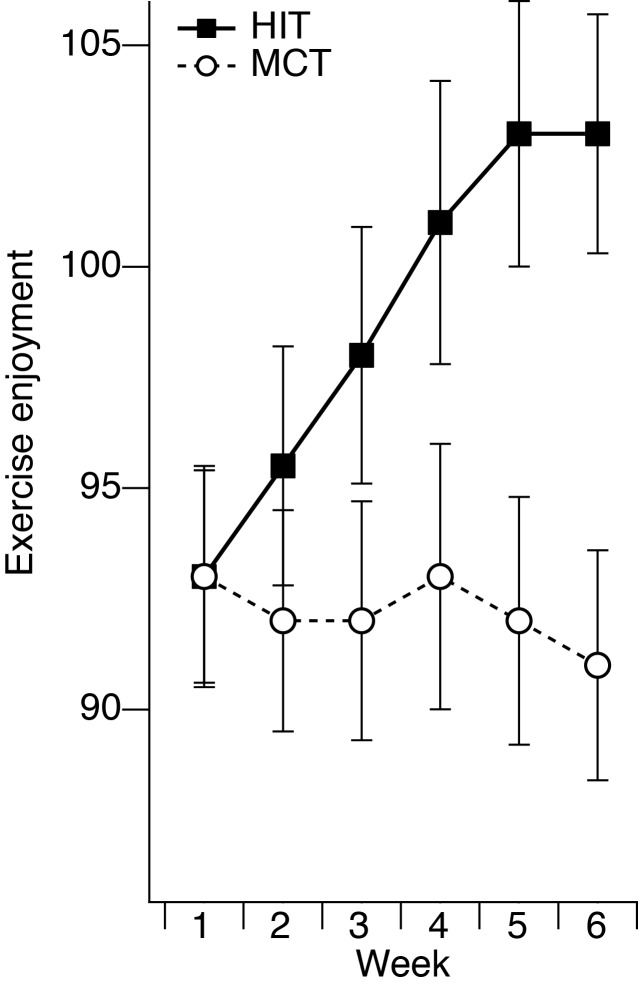
Mean exercise enjoyment across the first six weeks of high-intensity interval training (HIT) and moderate continuous training (MCT). Maximal score of enjoyment is 126, with higher scores indicating greater enjoyment. Error bars represent standard error of the mean.

Compared to MCT, HIT was rated as more enjoyable and the enjoyment for HIT progressively increased with training [Fig 1; main effect of week: *F*(5, 170) = 3.33, *p* < .01, *η*_*p*_^*2*^ = .09; interaction of group x week *F*(5, 170) = 3.54, *p* < .05, *η*_*p*_^*2*^ = .09; linear contrast of group x week: *F*(1, 34) = 6.73, *p* < .05, *η*_*p*_^*2*^ = 0.17]. Although groups did not differ in enjoyment after weeks one [*t*(34) = -0.02, *p* = .98], two [*t*(34) = 0.93, *p* = .36], or three [*t*(34) = 1.58, *p* = .13], HIT became marginal more enjoyable than MCT after week four [*t*(34) = 1.90, *p* = .07, *d* = .6] and was significantly more enjoyable after weeks five [*t*(34) = 2.48, *p* < .05, *d* = .9] and six [*t*(34) = 3.13, *p* < .01, *d* > 1].

### Change in enjoyment is predicted by change in wattage

Change in exercise enjoyment from weeks one-to-six was predicted by change in workload during that same interval, controlling for age and sex [Model 1: *F*(2, 33) = 3.54, *p* < .05; Model 2: *F*(3, 32) = 6.52, *p* < .01; *ΔF*(1, 32) = 10.46, *p* < .01; Table 4].

**Table 4:** Hierarchical regression analyses evaluated whether the change in exercise enjoyment was predicted by changes in exercise competency during the first six weeks of training.

	Beta	t	Partial correlation
Δ Workload (W)	0.62	3.23**	0.50
Δ PPO (W)	0.32	1.97#	0.33
Δ VO_2_ (ml/kg/min)	0.06	0.32	0.06

Peak power output (PPO)

#*p* = .06

**p < .01. Beta represents standardized coefficients.

## Discussion

The present study is the first to examine changes in enjoyment for high-intensity interval training (HIT) versus moderate continuous training (MCT) across the first six weeks of a training program. At the beginning of training, sedentary young adults in the HIT group reported similar levels of enjoyment to those in the MCT group. However, as training progressed, enjoyment for HIT increased whereas enjoyment for MCT remained constant and lower.

Differences in enjoyment for HIT over MCT were observed despite HIT being more physically strenuous. During an average training session, HIT elicited a higher workload, HR, and RPE than MCT but was shorter in duration to match protocols for energy expenditure. Even with the shorter time commitment, HIT induced similar physiological adaptations as indicated by pre-to-post change on maximal aerobic fitness test for both VO_2_ peak and PPO. Together, these results support the growing evidence that HIT is similarly effective but more time-efficient at improving aerobic fitness compared to more traditional moderate forms of continuous exercise [7, 17, 18]. Another important finding was that HIT and MCT caused non-significant reductions in resting-state/morning cortisol (Table 2). This may seem in contrast to the immediate increase in cortisol following an acute exercise bout, which is greater for high-intensity exercise than moderate-intensity exercise [19, 20]; however, the acute effect of exercise on cortisol is very transient and often return to baseline or slightly below resting-state levels following the end of the acute bout [21]. Taken together, these results suggest that despite the strenuousness of HIT, the training intensity did not impede exercise enjoyment or disrupt the baseline stress response during the initial six weeks of training.

The novel observation that training enhanced the enjoyment for HIT in sedentary young adults may help to clarify the equivocal findings regarding the enjoyment for acute bouts of HIT. Although an acute bout of high-intensity interval exercise is often rated as more enjoyable than an acute bout of moderate continuous exercise [4–6], enjoyment ratings are reduced when the high-intensity intervals are too strenuous or difficult to complete [8, 9]. Here, we used a HIT protocol that was physically challenging (as indicated by RPE, Table 3) and at the beginning of training our sedentary participants reported comparable enjoyment for HIT and MCT protocols. However, after four weeks of training, participants began to rate HIT as more enjoyable than MCT, and their enjoyment for HIT continued to increase until the end of the six weeks of training. The results suggest that chronic training may also lead to increased enjoyment for strenuous HIT protocols in sedentary young adults.

Competency with the exercise may be a key contributor to its enjoyment. Across the six weeks of training, increases in workload predicted increases in enjoyment. Similarly, greater increases in PPO achieved on the maximum aerobic test marginally predicted enjoyment whereas increases in VO_2_ peak did not. These results suggest that increased exercise enjoyment with training may be driven by physiological adaptations in strength rather than aerobic fitness, and this has important implications for identifying underlying physiological mechanisms.

However, the relationship between gains in strength and enjoyment may also be influenced by psychological factors. Specifically, participants were not blind to their training progress and could have easily tracked their progression in workload across each training session. Seeing workload increase across sessions would have provided positive feedback to increase motivation [22] and enjoyment for the exercise [11]. This may be why we did not observe a similar relationship between changes enjoyment and VO_2_; participants were not able to explicitly track their change in VO_2_ across sessions and therefore it was not as salient of a cue. Although both HIT and MCT groups would have received the same information with respect to increases in workload, positive performance feedback following an acute bout of exercise has been shown to increase enjoyment for high-intensity but not moderate-intensity exercise [11]. Although in the present study feedback was not explicit, this may be an important variable to control in future studies to dissociate the physiological and psychological contributions to the observed training-induced improvements in exercise enjoyment.

Incidental comparisons made with past personal experiences or with other participants within and across groups may have also influenced exercise enjoyment. However, given the random group assignment and low level of physical activity over the past three months for all participants, these potential influences would have been present from the start of the training and similar for both groups. Instead, HIT and MCT groups started off at the same level of exercise enjoyment in week 1 and only the enjoyment for HIT increased with training (Fig 1). This suggests that the gradual increase in enjoyment for HIT was directly related to the changes that took place during the six weeks as a consequence of HIT training.

The current examination of enjoyment during the first six week of a new exercise program provides critical information about exercise adoption and maintenance. Although participants in our study were incentivized to adhere to the program, in more naturalistic settings the initial six weeks represents a particularly vulnerable period for exercise adoption. When participants are free to withdraw from an exercise study without penalty, a steep dropout rate is observed within the first three months [23]. In one study that examined adherence to a 32-week training program, 64% of participants who eventually dropped out did so within the first 8 weeks of training [24]. It follows that exercise enjoyment during these initiation weeks may be especially important for preventing dropout and promoting long-term adherence. Future research should determine how long these enjoyment differences last and whether strength adaptations continue to predict enjoyment over longer periods of time. Furthermore, given the health benefits of exercise for the prevention and management of chronic conditions such as diabetes, obesity and cardiovascular disease, it would also be important to determine whether these populations would experience similar increases in HIT enjoyment with training.

In conclusion, the present study is the first to show increases in enjoyment for HIT within the first six weeks of training. Changes in workload predicted changes in exercise enjoyment, suggesting that strength adaptations might be important for increasing exercise enjoyment. The results point to HIT as a promising training protocol for increasing exercise enjoyment to promote exercise adherence in sedentary young adults.
